# The Developmental Emergence of the Mental Time-Line: Spatial and Numerical Distortion of Time Judgement

**DOI:** 10.1371/journal.pone.0130465

**Published:** 2015-07-02

**Authors:** Sylvie Droit-Volet, Jennifer Coull

**Affiliations:** 1 Université Clermont Auvergne, CNRS, UMR 6024, Clermont-Ferrand, France; 2 Laboratoire des Neurosciences Cognitives, Aix-Marseille Université, CNRS, UMR 7291, Marseille, France; Duke University, UNITED STATES

## Abstract

The perception of time is susceptible to distortion by factors such as attention, emotion, or even the physical properties of the stimulus to be timed. In adults, there is now evidence for a left-right spatial representation of time or “mental time-line”, in which short durations map to the left side of space, whereas long durations map to the right. We investigated the developmental trajectory of the mental time-line, by examining how spatial and numerical stimulus properties affect temporal bisection judgements in 3 groups of children (5, 8 or 10 year olds), as well as in adults. In contrast to previous developmental studies of the spatial representation of time, we manipulated spatial *position* (left-right) rather than spatial magnitude (distance) so as to pinpoint the age at which the mental time-line begins to influence the judgement of time. In addition, we manipulated spatial position symbolically, either directly, using left- or right-pointing arrows, or indirectly, using low (1) or high (9) digits. In adults and older children (10 year olds), the rightward arrow and the higher digit were judged to last longer. However, time judgements were unaffected by arrow direction and digits in the younger children. Therefore, the temporal distortions induced by symbolic representations of space (arrows) or number (digits) emerged with development, suggesting that the mental time-line is not derived from a primitive spatial representation of time but, rather, is the fruit of learning and is acquired around the age of 8-10 years old.

## Introduction

What is time? In the opening sentence of his book “The child’s conception of time”, Piaget [1] responded to this question by explaining that his goal was “to place the development of the idea of time in the kinetic context outside which this concept has no meaning. We are far too readily tempted to speak of intuitive ideas of time…as if time could be perceived and conceived apart from the entities or the events that fill it” (p. 1). Probably influenced by his encounter with Einstein in 1928 in Davos, Piaget aimed to demonstrate that the notion of time is “space in motion” (p. 2). As he later wrote, the hypothesis that he would like to defend is that psychologically time depends on “speed of movements” [2,3]. He thus designed a paradigm in which children had to judge the movement duration of two toy cars. Although both toys moved for the same amount of time, one moved faster and so stopped further away. Consistent with his hypothesis, children who had not yet attained the concrete operational stage of intellectual development, which would allow them to mentally coordinate speed and distance and, thereby, to infer time, were unable to judge that objects moving at different speeds could have the same duration. In the field of psychology, time was thus initially conceived as information derived from more primitive spatial information. However, the field then lost interest in the investigation of time *per se* or, as Block and Zakay [4] (p. 162) put it, “psychology loses time” between 1927 and 1958.

However, in the 1980s, there was renewed interest for the study of time stimulated by the results of rodent studies showing that animals devoid of sophisticated reasoning ability were able to accurately judge time [5,6,7]. Theories were then proposed, purporting the existence of an internal mechanism for measuring the passage of time (i.e. duration), the so-called “internal clock”. These theories initially suggested that the raw material for time representation is provided by a pacemaker-like mechanism that emits pulses during the stimulus to-be-timed [8,9,10]. The most recent versions of the internal clock, which aspire to be more biologically realistic, have replaced the idea of a pacemaker with that of temporal oscillators [11,12,13,14]. These temporal oscillators are single neurons, or sets of neurons distributed throughout the cortex, that fire at a regular frequency. Yet whatever the mechanisms, and corresponding output, involved in the representation of time, the predictions of the different internal clock models are similar at the behavioral level. The longer the objective time, the longer the temporal estimate will be, because a greater number of ticks (pulses, beats, oscillations, regular spikes) are accumulated [15,16]. Since these internal clock models were proposed, the main goal of many timing researchers has been to provide data to validate the theories’ predictions and to demonstrate that both animals and humans can accurately judge the passage of time [17].

Yet even though we are capable of accurately judging the duration of events, time judgments can nevertheless be distorted by certain contexts. This paradox was highlighted by Droit-Volet and Gil [18]: if we possess such a sophisticated mechanism for measuring time, why are our time estimates so variable? What clockmaker would keep such a clock? Several researchers have reopened the debate on the relationship between time and space, and cast doubt on the existence of a dedicated mechanism for time measurement in the brain [19,20]. For example, in 2008, Vicario, Oliveri and their colleagues asked participants to estimate the presentation duration of the digits “1” and “9” [21,22]. These digits were displayed in the center of the computer screen or on the right- or left-hand side. Participants judged the presentation duration of the digit 9 to be significantly longer than that of the digit 1, confirming earlier observations [23,24,25] that numerical magnitude interfered with time perception [26]. In addition, irrespective of the magnitude of stimuli to be timed, the spatial location of stimuli affected temporal judgments, with stimuli being judged longer when presented on the right- than the left-hand side [22]. The authors concluded that these lengthening effects are due to time being internally mapped onto a spatial representation, a “mental time-line” [20], just as number is thought to spatially map on to a “mental number line” [27,28,29].

In 2008, several other studies provided further support for this idea using reaction time (RT) paradigms. For example, Weger and Pratt [30] found that RTs to stimuli representing the past or the future were faster when presented on the left or right-hand side of the screen, respectively. Similarly, response speeds to targets appearing after short or long intervals [31] or targets appearing earlier or later than expected [32], or for durations judged to be shorter or longer than a standard [33] depended upon the spatial compatibility of stimulus-response mappings: RTs were faster when “short” trials were responded to with a left-, rather than right-, sided response, whereas RTs were faster for “long” trials when responses were right-, rather than left-, sided. This is the temporal equivalent of the “SNARC” (Spatial Numerical Association of Response Codes) effect [27], whereby RTs are faster for small numbers when responded to with a left-sided response, but for large numbers when responded to with a right-sided response. Such results have been used to defend the theory of a common system for the processing of all magnitudes [19,21,22,30]—including time, space and number–which is based on spatial coordinates with a left-right oriented representation.

The interfering effects of space and number on time judgment have been taken as evidence for an internal spatial representation of time, as previously suggested by Piaget [1]. However, these studies examined the influence of spatial and numerical information on time judgment in adults who had already acquired an abstract concept of time (Newtonian time), in which time is represented along a spatial continuum going from left to right. Time is indeed conceived in Western culture as a left-to-right mental time line [34,35], with the direction being reversed [36] or rotated to the vertical axis [37] in other cultures. These empirical results support the colloquial notion of “time’s arrow” [38]. However, the question raised is this: are the spatial effects on time judgment rooted in a representation of time that is fundamentally spatial in nature, or do they result from a culturally acquired concept of a “mental time line” that acts upon an “internal clock”? It is indeed possible that participants have a dedicated clock system for processing time, but that different sources of non-temporal information can interfere with their time judgments through non-spatial (e.g. attentional) mechanisms. Consistent with this, results have often demonstrated an asymmetric relationship between the mutual influence of space and number on one hand, with time on the other. For example, in numerical and temporal bisection tasks, Droit-Volet et al. [23] showed that the processing of numerosity interfered with temporal judgments while the processing of time did not interfere with numerical judgments. Dormal et al. [24] further confirmed this asymmetric pattern in adults, using a Stroop interference paradigm (see also [39]. A similarly asymmetric relationship exists between space and time, with time judgements being influenced by concurrent spatial information, but not vice versa [40,41,42]. According to Casasanto and Boroditsky (2008), this asymmetry is inconsistent with the hypothesis of a common magnitude processing system but, instead, reflects an influence of how people *think* about time [40,43]. Indeed, people think and talk about time using spatial representations and many linguistic metaphors about time are spatial (e.g. time-travel) [44,45,46,47,48,49]. From this perspective, it is possible that young children who have not yet acquired a spatial representation of time would not be subject to spatial interference of time judgments.

Casasanto, Fotakopoulou and Boroditsky [50], and later Bottini and Casasanto [51], investigated the mutual influence of temporal and spatial information on duration and distance judgements in children. They replicated the asymmetric pattern of influence reported previously for adults [40] in young children (5 year olds), with space affecting time more than time affecting space. According to these authors, this suggests that young children had already acquired a spatial representation of time. However, these studies examined the influence of *spatial magnitude* on time (longer stimulus length led to longer estimates of duration) not the influence of *spatial position*, which is the aspect of spatial processing involved in representing time in the form of a left-right oriented mental time-line. It is still unknown whether children’s estimates of time would be similarly biased by *spatial position*. In other words, can we see evidence of a mental time-line in children?

The aim of our study therefore, was to examine how a left-right oriented stimulus might interfere with time judgments in children aged from 5 to 10 years old, i.e., during the developmental period in which children acquire a concept of time [52], and to compare this with the pattern of interference observed in adults. In the first experiment (Arrow), we examined this directly by presenting stimuli in the form of either a leftward or a rightward arrow, and comparing their effects on time bisection judgments. Indeed, to underline our interest in the influence of a *mentalised* representation of space on time judgements, we presented stimuli in the form of directional arrows, which were displayed in the center of the computer screen. This approach contrasts with paradigms in which stimuli physically appeared on the left or right hand side of the screen [22,30]. In the next two Experiments (Number), we examined this more indirectly, by measuring the influence of numerical magnitude on time bisection judgments in children and adults, using the same procedure as those used by Vicario et al. [22] and Oliveri et al. [21]. Our hypothesis was that if the left-right spatial representation of both time and number is “innate”, we should find similar patterns of spatial and numerical interference on time judgment across age groups. Alternatively, if it is due to the acquisition of a left-right spatial representation of time then time-related distortions would emerge during childhood, at an age when children master the concept of a mental time line.

## Experiment 1 –Arrow

### Method

#### Participants

163 subjects participated in this experiment: Forty 5-year-olds (mean age = 5.25, SD = 0.47, 20 girls and 20 boys), forty-three 8-year-olds (mean age = 7.75, SD = 0.33, 24 girls and 19 boys), forty 10-year-olds (mean age = 9.96, SD = 0.68, 21 girls, 19 boys), and forty adults (mean age = 21.33, SD = 2.58, 26 women and 14 men). The children were recruited from nursery and primary schools in Gerzat, France. In their classroom, and with the teacher's consent, the experimenter asked children (orally) whether they would like to voluntarily participate in a study. The adults were undergraduate students from the University of Clermont and signed a written informed consent. Parents signed the written informed consent on behalf of their children. The study was carried out according to the principles of the Helsinki Declaration. This study and the consent procedure for the children was approved by both the inspector of the academy of the French National Education Ministry and the Clermont-Ferrand Sud-Est VI Statutory Ethics Committee (CPP, Sud-Est 6, France) according to the articles of law L. 1121-1- 1 and R 1121–3. The students received course credit in exchange for their participation.

#### Material

The children were tested individually in a quiet room in their school. A computer controlled the experimental events and recorded data using E-prime (Psychology Software tools Inc.). Responses were made on the D and K keys of the computer keyboard, with the sticker “Short” or “Long” being placed on the relevant keys. Response contingencies were counterbalanced across participants. Three stimuli were used, all composed of the same small rectangle and triangle. For the rightward arrow, the triangle was on the right of the rectangle. For the leftward arrow, it was on the left of the rectangle, i.e., pointing in the opposite direction. For the control stimulus, used during the training phase only, the triangle was placed either above or below the rectangle, the triangle position changing randomly between trials. All stimuli were presented in the center of the computer screen.

#### Procedure

All participants performed a temporal bisection task in two successive sessions, each composed of a training and a testing phase. In the training phase, the participants were initially presented with 3 repetitions each of the short and the long standard durations, displayed in the form of the control stimulus. Participants were trained to press the “short” and “long” response keys after the short and the long standard durations respectively, over 8 trials (4 short, 4 long). Each trial started with the word “prêt” (“ready”) displayed in the center of the computer screen. When the participant was ready, the experimenter pressed the spacebar and the stimulus appeared in the center of the screen, for one of the two standard durations. Inter-trial intervals were randomly chosen between 0.5 and 1 s. In the testing phase that followed the training phase, participants were presented with the rightward (→) or leftward arrow stimulus (←), in the center of the screen, for each of seven comparison durations (i.e. a block of 14 randomized trials). They performed 5 blocks of 14 trials in each of the two testing sessions. The total number of trials over 2 sessions was thus 140 trials. In addition, the participants were assigned to one of two duration-range groups as a function of the duration range of the comparison stimulus. In the 200/800-ms condition, the short and the long standard durations were 200 ms and 800 ms and the comparison durations were 200, 300, 400, 500, 600, 700 and 800 ms. In the 400/1600-ms condition, the standard durations were 400 and 16000 ms, and the comparison durations 400, 600, 800, 1000, 1200, 1400, and 1600 ms.

### Results and Discussion

**Fig 1:**
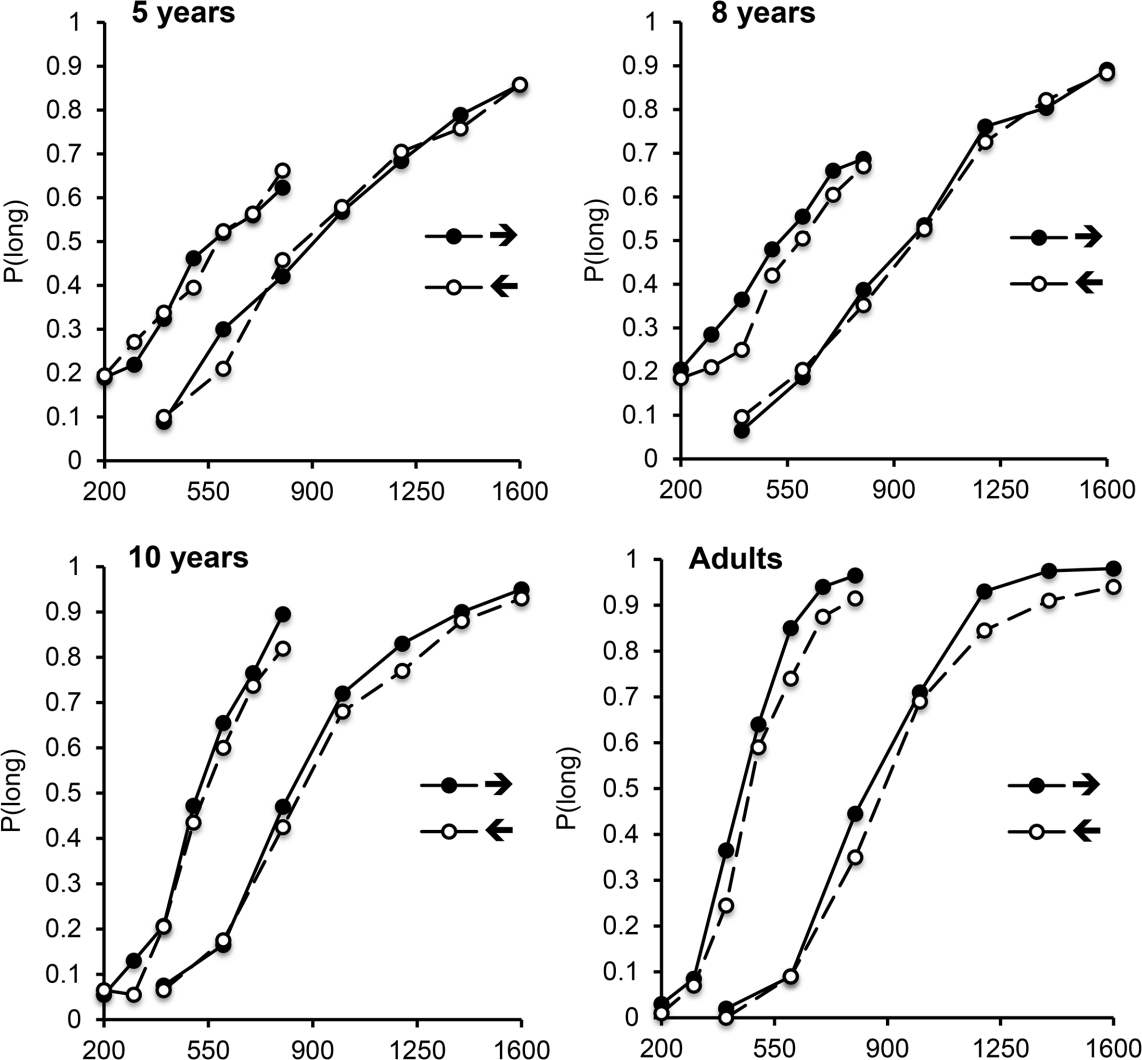
Bisection functions in “Arrow” Experiment. Proportion of long responses plotted against comparison durations for the rightward arrow (→ the leftward arrow (←) for each age group, in the 200/800 and the 400/1600-ms duration conditions.

Fig 1 shows the mean proportion of long responses (*p*(long)) plotted against comparison duration (i.e. the psychophysical functions) for the rightward (→) and leftward arrow (←) in each of the 4 age groups. A shift in the curve to the left indicates longer duration judgements. Inspection of Fig 1 suggests that the shift in the bisection curve to the left for the rightward, compared to leftward, arrow, emerged between 8 and 10 years old, irrespective of the duration range tested. Initial ANOVAs that included response-side as a factor were conducted on the different temporal indices used in our study (*p*(long), BP, WR). For each age group, the bisection task results systematically revealed neither main effect of response-side nor interaction involving this factor (all *p* > .05). This factor was thus excluded from subsequent statistical analyses. The ANOVA on *p*(long) with 2 within-subject (arrow, comparison duration) and 1 between-subjects (duration range) factor for each age group confirmed that the effect of arrow was not significant in the 5-year-old children, *F*(1, 38) = 0.01, *p* = .99, nor in the 8-year-old children, *F*(1, 41) = 1.20, *p* = .28. By contrast, the 10-year-old children and the adults judged the presentation duration of the rightward arrow to be longer than that for the leftward arrow (10 years: .52 *vs*. .49, *F*(1, 38) = 7.94, *p* = .01, η2 = .17; Adults: .57 vs. 52, *F*(1, 38) = 21.19, *p* = .0001, η2 = .36). The arrow factor did not interact with any other factors in any age group (all *p* > .05).

For the 5- and 8-year-olds, only the main effects of comparison duration [*F*(6, 228) = 97.18, *p* = .0001, η2 = .72; *F*(6, 246) = 121,68, η2 = .75, respectively], duration range [*F*(1, 38) = 8.24, *p* = .01, η2 = .18, *F*(1, 41) = 4.19, η2 = .09], and their interaction [*F*(6, 228) = 6.57, *p* = .0001, η2 = .15, *F*(6, 246) = 8.03, η2 = .16] were significant (all *p* < .05). The main effects of comparison duration and duration range, as well as their interaction, were also significant in the 10-year-olds [*F*(6, 228) = 255.75, η2 = .87; *F*(1, 38) = 15.24, η2 = .29, *F*(6, 228) = 4.50, η2 = .11, respectively, all *p* < .05], while only the main effect of comparison duration reached significance in the adults [comparison duration, *F*(6, 228) = 461.66, *p* = .0001, η2 = .93, duration range, *F*(1, 38) = 2.05, comparison duration x duration range, *F*(6, 228) = 1.34, both *p* > .05]. In sum, the main effect of duration obtained across all age groups confirmed that *p*(long) increased with stimulus duration, consistent with the ability of children as young as 5 to discriminate time [53,54]. Moreover, the dissociation in the effects of arrow direction between groups demonstrated that participants responded “long” significantly more often for the rightward arrow (→ than for the leftward arrow (←) only from the age of 10 years old.

We further analyzed the results by deriving the Bisection Point (BP) and the Weber Ratio (WR) from the fit of the pseudo-logistic function [55] to individual psychophysical functions. The fit of this function was indeed significant for most participants (5 years, mean R^2^ = .83, SD = .13; 8 years, mean R^2^ = .90, SD = .08; 10 years, mean R^2^ = .91, SD = .09; Adults, mean R^2^ = .96, SD = .03; all *p* < .05). However, it was not significant for a proportion of the children, all of whom were in the 200/800-ms group (5 years olds, N = 8; 8 year-olds, N = 10; 10 years, N = 1). Their results were thus excluded from subsequent statistical analyses. The BP is the point of subjective equality, i.e. the stimulus for which the participants respond long as often as short (*p*(long) = .05): the lower the BP, the longer the subjective time estimate. The WR is a measure of time sensitivity (Difference limen ([*p*(long) = .75—*p*(long) = .25]/2) divided by the BP): the lower the WR value, the greater the time sensitivity and the steeper the psychophysical function. The ANOVAs run on the WR showed neither effect of arrow nor interaction with this factor in any age group (all *p* > .05) (Table 1). Consequently, arrow direction did not affect sensitivity to time. The duration range effect was also not significant (all *p* > .05) indicating a constant Weber Ratio for the different duration ranges consistent with the scalar properties of time.

**Table 1:** Weber Ratio in the “Arrow Experiment”.

	5 years		8 years		10 years		Adults	
Conditions	M	SE	M	SE	M	SE	M	SE
**200/800-ms condition**								
→	0.63	0.08	0.30	0.08	0.26	0.06	0.16	0.06
←	0.73	0.07	0.30	0.08	0.26	0.06	0.17	0.06
**400/1600-ms condition**								
→	0.49	0.06	0.30	0.06	0.22	0.06	0.15	0.06
←	0.39	0.06	0.27	0.08	0.23	0.06	0.17	0.06

Mean (M) and standard error (SE) of the Weber Ratio for the rightward (→ and the leftward (←) arrow for each age group in the 200/800 and the 400/1600-ms duration conditions

**Fig 2:**
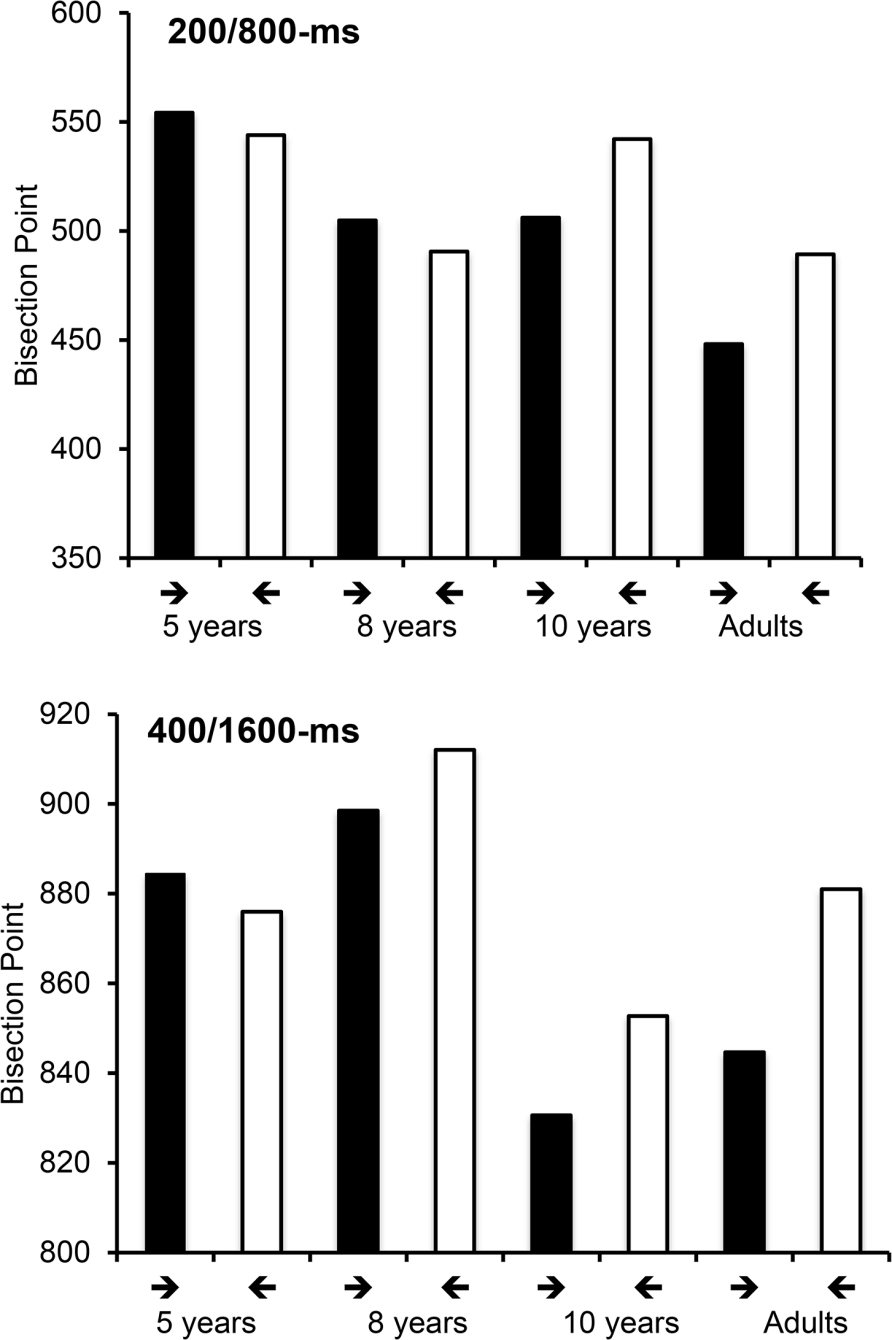
Bisection Point in “Arrow Experiment”. Bisection point for the rightward arrow (→ the leftward arrow (←) for each age group, in the 200/800 and the 400/1600-ms duration conditions.

More interesting for the purpose of this study, the ANOVAs of the BP showed a significant main effect of arrow for the adults and the 10-year-old children (*F*(1, 38) = 11.17, *p* = .001, η2 = .23, *F*(1, 37) = 4.35, η2 = .11, respectively, p < .05), but not for the 8-year-olds, nor the 5-year-olds, *F*(1, 31) = 0.001, *F*(1, 30) = 0.11, *p* > .05), which confirms the main effect of arrow on *p*(long), described above. The arrow effect did not interact with duration range (all *p* > .05), although the duration range effect was systematically significant (adults: *F*(1, 38) = 127.43, η2 = .77; 10 years: *F*(1, 37) = 39.14, η2 = .52; 8 years *F*(1, 31) = 27,77, η2 = .47; 5 years, *F*(1, 30) = 25.06, η2 = .46, all *p =* .*001*), with BP values being longer in the 400/1600-ms than in the 200/800-s duration range. As illustrated in Fig 2, the BP was significantly lower for the stimulus featuring the rightward arrow than the leftward arrow, but only for the two oldest age groups. The magnitude of the difference in the BP between these 2 stimuli was similar between the 10-year-olds and the adults, *t*(77) = .55, *p* = .58. In other words, the lengthening effect observed for the rightward arrow did not appear at an early age, but emerged between 8 and 10 years old.

These results suggest this phenomenon depends on acquisition of a left to right mental time-line that interferes with time judgment. We tested this hypothesis further, by exploring the potentially interfering effects of number on time judgements using the bisection procedure initially employed by Vicario et al. [22], with digits 1 and 9 in the testing phase and the digit 5 as a reference in the training phase. Processing of number is thought to share a common magnitude representation with time, as well as space [56]. Consequently, if number is represented spatially in a left to right direction [22,27], then the influence of number on time judgements should show a similar developmental trajectory to that for the influence of spatial position (i.e., rightward and leftward arrow).

## Experiment 2—Number

### Experiment 2.1: Method

#### Participants

Eighty new children participated in this Experiment: Twenty 5-year-olds (mean age = 5.04, SD = 0.43, 12 girls and 8 boys), twenty 8-year-olds (mean age = 7.8, SD = 0.36, 14 girls and 6 boys), twenty 10-year-olds (mean age = 10.33, SD = 0.48, 9 girls and 11 boys), and twenty adults (mean age = 20.56, SD = 1.89, 13 women and 7 men). The children were recruited from nursery and primary schools in Clermont-Ferrand and the adults were undergraduate students at the University of Clermont. Following the consent procedure described in Experiment 1, the children’s parents and the students signed a written informed consent for this study, which was approved both by the inspector of the academy of the French National Education Ministry and the Statutory Ethics Committee (CPP, Sud-Est 6, France) according to the articles of law L. 1121-1- 1 and R 1121–3. The students also received course credit.

#### Material and Procedure

The materials and procedure used were similar to those described in Experiment 1, with stimuli being presented in the center of the computer screen. Only the form of the stimulus to-be-timed changed, being now one of 3 different digits: 1, 5 or 9. The participants performed a temporal bisection task with stimulus durations of 200-ms and 800-ms for the short and the long standard durations, respectively, and comparison durations of 200, 300, 400, 500, 600, 700 and 800 ms. As for the previous experiment, the bisection task was given over 2 successive sessions, each composed of the same training and testing phases. In the training phase, the short and the long standard durations were presented in the form of the digit 5. In the testing phase, the stimuli used for comparison durations were the digits 1 and 9. Response contingencies were also counterbalanced across participants. The participants were given 5 blocks of 14 randomized trials. Each block consisted of one trial for the digit 1 and another for the digit 9, for each of the 7 comparison durations. With the 2 sessions, this made a total of 140 trials (5 x 2 x 7 x 2).

### Results and Discussion

**Fig 3:**
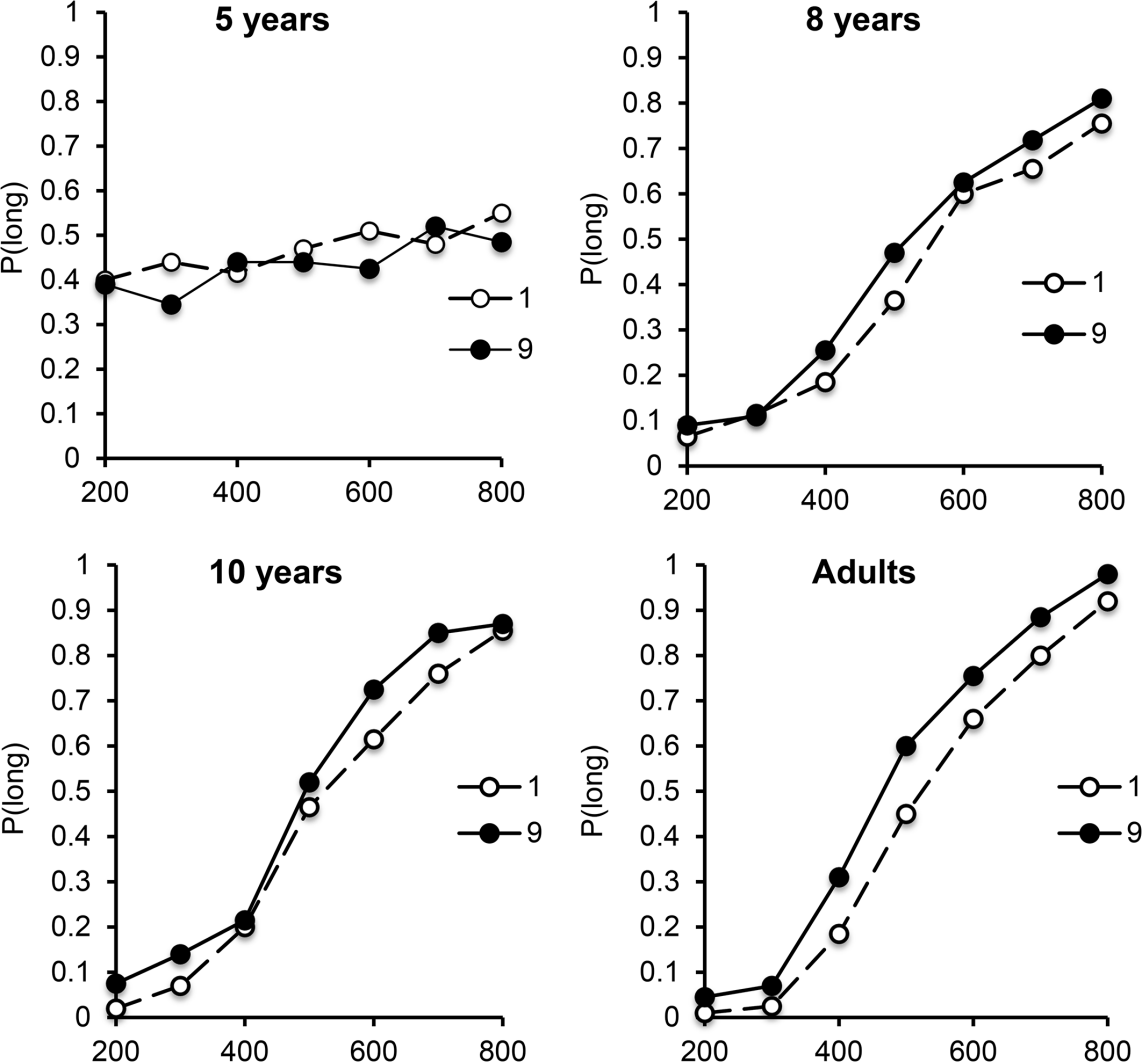
Bisection functions in “Number Experiment”. Proportion of long responses plotted against comparison durations for the number 1 and 9 for each age group, in the 200/800-ms duration condition.

In this bisection study, no significant effect involving the response-side factor was found for any age group (all, *p* > .05). This factor was therefore excluded from further statistical analyses. Fig 3 shows the psychophysical functions obtained for the digits 1 and 9 in each of the 4 age groups. Inspection of this figure clearly shows a shift in the psychophysical function toward the left for age groups older than 5 years old, indicating that presentation duration was judged to be longer for the number 9 than for the number 1. In fact, the presentation of stimuli in the form of two different digits in the testing phase totally disrupted temporal performance in the youngest children, as revealed by their flat psychophysical functions for both the number 1 and the number 9 stimuli. For the 5-year-olds, the ANOVA on *p*(long) nevertheless showed a main effect of comparison duration, *F*(6, 114) = 3.55, η2 = .16, *p* = .003, but the digit effect and the comparison duration x digit interaction were not significant (*F*(1, 19) = .70, *F*(6, 114) = 1.18, all *p* > .05). In contrast, the digit effect was significant for the older age groups (8 years, *F*(1, 19) = 4.40, η2 = .19, *p* = .05; 10 years, *F*(1, 19) = 9.03, η2 = .32, *p* = .007; adults, *F*(1, 19) = 20.11, η2 = .52, *p* = .0001). In these age groups, digit did not interact with comparison duration (*F*(6, 114) = .95, *F*(6, 114) = 1.14, *F*(6, 114) = 1.55, all *p* > .05), although comparison duration was systematically significant (*F*(6, 114) = 127.99, η2 = .87, *F*(6, 114) = 115.38, η2 = .86, *F*(6, 114) = 253.79, η2 = .93, all *p* = .0001).

**Fig 4:**
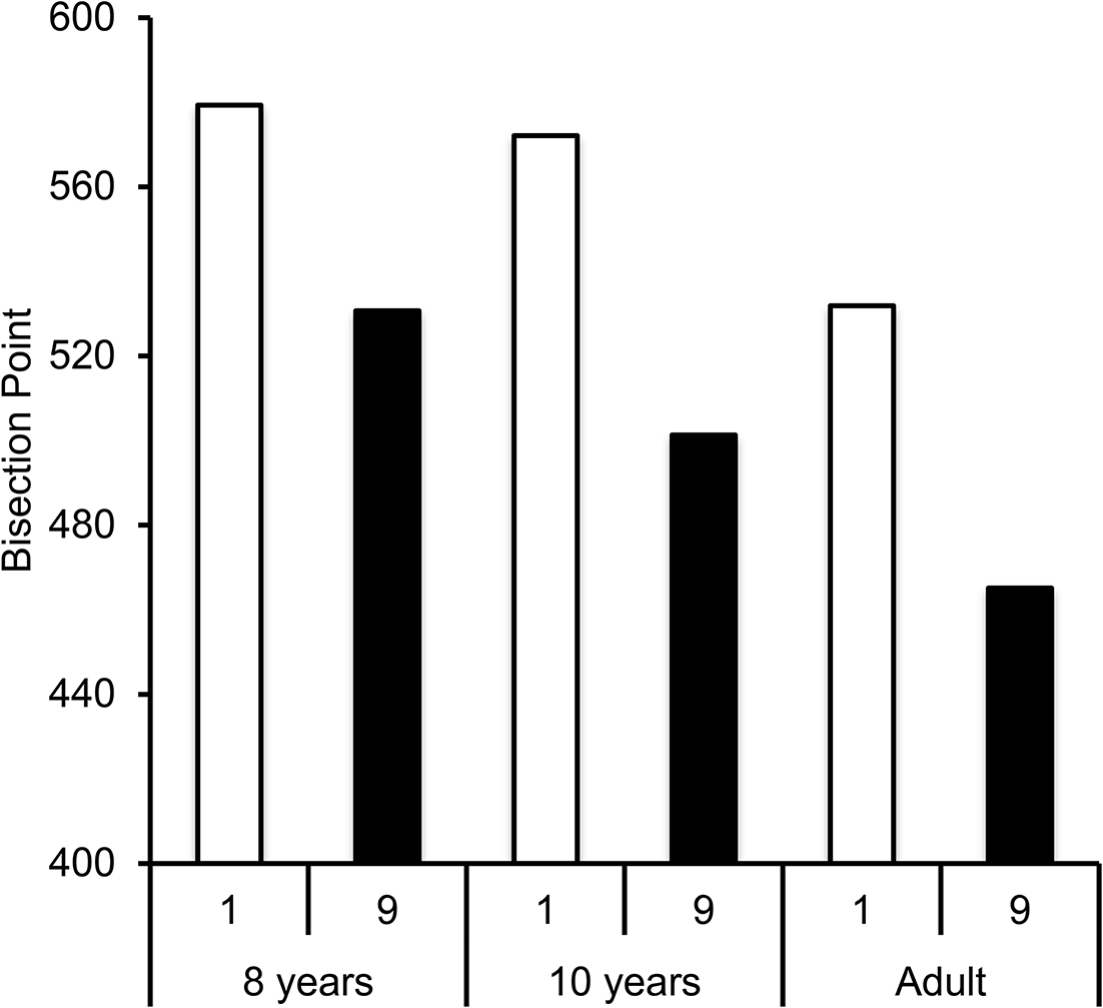
Bisection Point in “Number Experiment”. Bisection point for the number 1 and 9 for each age group, in the 200/800-ms duration condition with the same standard duration presented in the form of the digit 5.

To further examine our bisection results, we measured a BP and a WR for each experimental condition, by fitting a pseudo-logistic function to individual data (8 years, R^2^ = .87; 10 years, R^2^ = .93; adults, R^2^ = .95, all *p* < .05). The flat bisection curves for the 5-year-olds were obviously excluded from these analyses. The fit was not significant for one 10-year-old whose results were also excluded from the subsequent statistical analyses. The ANOVA on the BP showed a significant main effect of digit, *F*(1, 56) = 10.74, η2 = .52, *p* = .002, such that BP was lower for the number 9 than for the number 1 (Fig 4), which is consistent with a lengthening effect of large numbers. Surprisingly enough, the effect of digit also reached significance for the WR, *F*(1, 56) = 4.47, η2 = .07, *p* = .04, suggesting a tendency for participants to be more variable in their time judgments for the high than low digit. There were no significant interactions between digit and age for either the BP or WR, suggesting that effects were similar for the 8 year-olds, 10 year-olds and adults.

In sum, our results demonstrated that symbolic representations of numerical magnitude interfered with temporal bisection performance by producing a lengthening effect on time judgement, such that the presentation duration of the higher number (9) was judged to last longer than that of lower number (1). This interference was so strong that it prevented the youngest children from being able to discriminate time at all. Yet, numerous studies have already shown that 5-year-old children are capable of discriminating different durations [e.g. 57]. Consequently, to further investigate the effect of digits on time perception in the youngest children, we decided to run a new experiment with 5-year-olds, using the same digits as in Experiment 2.1 (1, 9), but now these digits were not randomly intermixed within the same testing phase but presented independently and successively in different testing phases with the digit 5 always presented as reference in the training phase. We hypothesized that presenting different digits in discrete blocks would minimize the interfering effects of automatic number processing in these young children, allowing them to focus instead on the timing task. We aimed to make the task simple enough to allow psychophysical functions to be computed which would, in turn, allow putative effects of digit processing on temporal judgements to be measured.

### Experiment 2.2: Method

#### Participants

Nineteen new 5-year-old children participated in this experiment (mean age = 5.16, SD = 0.41, 7 girls and 12 boys). One additional child began the experiment but then withdrew. The children came from another nursery school in Clermont-Ferrand. As for the other experiments, parents signed the written informed consent for participation of their children in this study. The experimenter asked (orally) for volunteers for the study in the children’s classroom, with the teacher’s consent. This study, with its consent procedure, was approved by the inspector of the academy of the French National Education Ministry and the Ethics Committee (CPP, Sud-Est 6, France).

#### Material and Procedure

The material was the same as that used in Experiment 2.1, with the standard durations always presented in the form of the digit “5”. The children were given 3 successive testing phases of 56 trials: 8 trials for each of the 7 comparison durations (200, 300, 400, 500, 600, 700, 800). This made a total of 168 trials (3 x 8 x 7). The digit “5” was presented in the first testing phase, the digit “1” in the second testing phase and the digit “9” in the third testing phase. Before each testing phase, the children were given a training phase with the short (200-ms) and the long (800-ms) standard durations, presented in the form of the digit “5”.

### Results and Discussion

**Fig 5:**
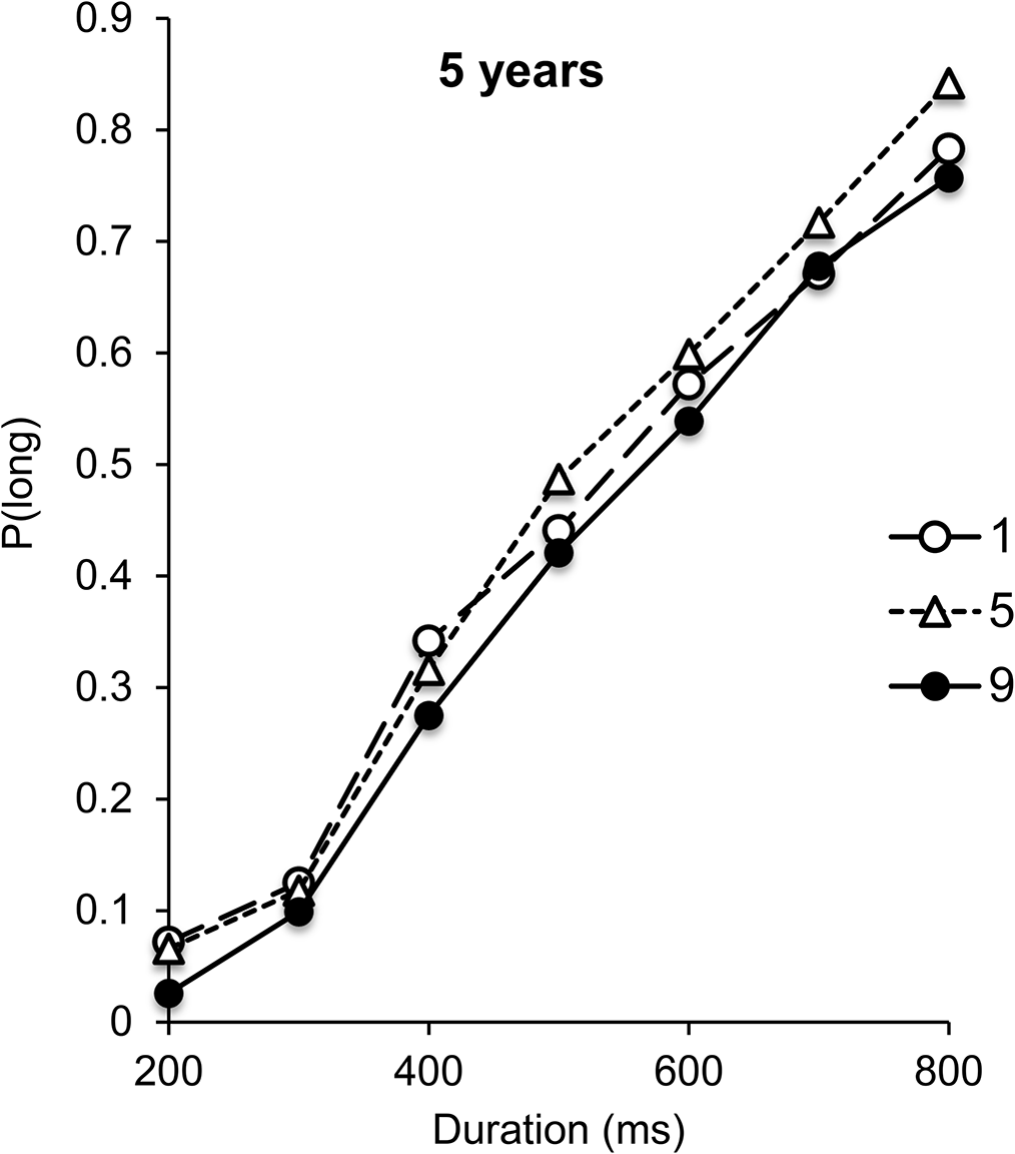
Bisection functions for the 5-year-olds in “Number Experiment”. Proportion of long responses plotted against comparison durations for the number 1, 5 and 9 for the 5-year-old children in the 200/800-ms duration condition, when the numbers were presented on separate blocks of trials with the same standard durations presented in the form of the digit 5.

Fig 5 shows the bisection curves obtained by the 5-year-olds in the different testing phases with the digits 1, 5 and 9. As in the other studies, response-side was not included in the statistical analyses because the initial ANOVA did not show any significant effect involving the response-side factor (*p* > .05). When different digits were not randomly intermixed within the same testing phase, these young children succeeded in discriminating stimulus duration, whatever the digit presented. This was confirmed by statistical analyses on *p*(long), which showed a significant main effect of comparison duration, *F*(6, 108) = 97.47, η2 = .84, *p* = .0001. However, a main effect of digit still did not appear for these 5-year-olds, *F*(2, 36) = 1.35, *p* = .27, nor a digit x comparison duration interaction, *F*(12, 216) = 0.39, *p* = .97. This was confirmed by analysis of the BP and WR, which, as in the previous experiments, were derived from the significant fit of the pseudo-logistic function to individual data (R^2^ = .88, SD = .06, *p* < .05) (this fit was not significant for 2 children, who were excluded from the analysis). The digit effect was not significant for the BP (1-digit: 506; 5-digit: 482; 9-digit: 527, *F*(2, 32) = 1.35, *p* = .27), nor the WR (1-digit: 0.36, 5-digit: 0.33; 9-digit: 0.29, *F*(2, 32) = .50, *p* = .61), even though pseudo-logistic functions were successfully fitted to the majority of the 5-year-olds’ data. Unfortunately, we did not counterbalance the order of the digit conditions, meaning that any significant shifts in temporal performance could have been interpreted as order effects, rather than digit-induced biases. However, there were no significant differences in performance across the three different blocks, suggesting that even if data were driven by order effects rather than digit effects, they did not significantly influence behaviour. In sum, despite the fact that the standard duration in reference memory was encoded with the digit 5 during the training phase, the presentation of higher or lower digits during the testing phase did not distort time judgments in 5-year-olds. In other words, the interfering effects of a symbolic representation of number on the perception of time, which produced a lengthening effect for the digit 9 in older children and adults, was not present for the youngest age group. Taken together, the results of our experiments on number showed interfering effects of digit stimuli on time judgment, although the lengthening effect observed in adults [22] and older children did not emerge in the youngest children’s temporal judgements.

## General Discussion

Our results confirmed that, in adults, the spatial representation of stimuli interfered with judgments of their presentation duration. Indeed, the results of Experiment 1, using arrows as stimuli, showed that adults responded “long” more often for the rightward arrow than for the leftward arrow and that this significantly lowered the bisection point. These results are consistent with Vicario et al.’s findings in adults of a time dilation effect for a right versus left spatial representation [22]. However, Vicario et al. [22] presented stimuli physically on the right- and left-hand side of the computer screen, whereas we used directional arrow stimuli, which provide a more symbolic representation of space. This suggests, therefore, that the spatial representation of time (mental time-line) influences adult’s judgements of time whether spatial information is represented physically or more symbolically.

The main novelty of our study however, was to test for the presence of these temporal distortion effects in children of different ages. We found that, like adults, 10-year-olds’ time judgements were biased by the direction of the arrow. Indeed, at 10 years old, the children judged the rightward arrow to last longer than the leftward arrow. Crucially, however, the 5- and 8-year-olds’ time judgments were not affected by arrow direction. This result is unlikely to simply reflect an inability of younger children to correctly interpret the arrow. Cued reaction time (RT) studies have shown that the spatial position indicated by an arrow stimulus is processed automatically not only by adults [58, 59] but also by children as young as 3–5 years of age [60]. In these studies, the experimental procedure is defined so that arrow cues are non-predictive of target location: the target is as likely to appear in the location indicated by the arrow, as not. Moreover, participants are explicitly told that arrow direction will not predict target location. Nevertheless, RTs are faster for targets appearing in the location indicated by the arrow, suggesting that preschool children automatically process arrow direction and use it to modify their performance. This is most likely because the perceptual weight of the arrowhead on one side of the screen or other is cueing spatial attention in a bottom-up, exogenous manner. Moreover, even when the perceptual properties of arrow stimuli are controlled for, 5 year olds are still capable of using the conceptual meaning of arrows to guide performance in a more top-down endogenous manner [61].

The fact that the directional arrow effect was not observed across all age groups but emerged with development suggests the left-right representation of time, i.e. the “mental time-line”, is not derived from a primitive spatial representation of time that is automatically activated, but, rather, is the fruit of learning. Consistent with this idea, our study showed that the arrow-induced distortion of time judgements emerged between about 8 and 10 years old, which is when children begin to logically reason about time [1,52]. Throughout the world, people learn to communicate and think about time using spatial representations [37]. This way of thinking about time might thus influence their perception of time. In support of this, Lamotte, Droit-Volet and Izaute [62] and Droit-Volet, Lamotte and Izaute [63] showed that simply being aware of subjective time distortion modifies empirical judgements of time. Consequently, the results of our study suggest that acquisition of a left-to-right spatial representation of time throughout childhood, whether through acquisition of culture-specific linguistic metaphors [37,44] or reading and writing habits [20,36], leads to biases in time judgment. Evidence that the spatial representation of time in terms of the mental time-line is acquired, rather than innate, is further bolstered by studies showing that, in adults, training can modify, or even reverse, the spatial (and numerical) influence on time judgement [46,50,64,65]. However, very recently, Rugani et al. [66] have shown that even 3-day old chicks represent number in terms of spatial position, spontaneously associating smaller numbers with the left- and larger numbers with the right-side of space. Consequently, it is possible that the mental time-line becomes spatially mapped during the course of development, whereas number is spatially mapped from birth.

In further support of our conclusion that a left-right spatial representation of time is acquired thoughout childhood, we found that the influence of numerical magnitude on time judgement was absent in 5-year-olds, despite being present in adults, 10-year-olds and 8-year-olds. It was only from the age of 8 years old that participants judged the presentation time of the digit 9 to be longer than that of the digit 1. This is unlikely simply to reflect 5 year olds inability to interpret digits since children in this age group understand the numerical representation of digit stimuli [67]. However, results from the numerical Stroop task [68–71] and priming paradigms [72] indicate that automatic processing of digit stimuli occurs only from the age of 5 to 6 years old. Therefore, it’s possible that the 5 year olds in our study may have simply been ignoring the fact that stimuli to be timed were presented in the form of digits. However, our study revealed that the presentation of stimuli in the form of digits strongly interfered with 5 year olds temporal performance, preventing them from accurately processing time at all and rendering their psychophysical functions flat (Experiment 2.1). Such interference suggests that even though number was irrelevant to the timing task the youngest children were nevertheless processing the digit stimuli automatically (see also [72]). In a follow-up experiment, with another group of 5 year olds, we reduced the number-related interference effect by presenting each digit (1 or 9) in separate blocks of testing trials, which helped young children to accurately judge stimulus duration and allowed us to calculate psychometric functions from individual data (Experiment 2.2). Nevertheless, the magnitude of the test digit (1 or 9) still had no effect on the relative length of 5 year olds’ time judgements. Using the same bisection procedure, Vicario et al. [22] argued that the interference of spatial position and numerical magnitude on time judgment supports a common system of representation for all magnitudes—time, space, number—based on left-right oriented spatial coordinates. However, our results demonstrated that although salient non-temporal information (i.e., number) interfered with temporal processing early in childhood, possibly via attentional mechanisms, the lengthening effect of number on time judgement only emerged later in development (around 8 years old), at least in the case of numbers presented in the form of digits. As suggested previously, this is unlikely to be simply because 5 year olds did not automatically process the digit stimuli. Using a simplified version of the SNARC paradigm, in which digit magnitude was irrelevant for task performance and so accessed *automatic* spatial representation of number, Hoffmann et al. [73] found a SNARC effect (faster RTs for left/right hand responses to small/large numbers) in children as young as 5. Interestingly, this contrasts with studies of the classic SNARC paradigm, in which participants must make explicit judgements of digit magnitude, showing that the SNARC effect appears later, around 7–8 years of age [74,75]. Therefore, the age at which we found number begins to affect explicit time judgements (age 8) is approximately the same as the age at which space begins to affect explicit number judgements. This suggests that the developmental emergence of the mental time-line may be linked to the acquisition of an explicit spatial representation of number.

Our data also suggest that time judgements are disrupted by number more than spatial position, with 5-year-olds’ timing performance being entirely disrupted by presentation of stimuli in the form of digits, but not arrows. This supports findings in adults that the interfering effects of number on time perception are greater than those of space [41]. An alternative, though not mutually exclusive, interpretation of this dissociation is that we manipulated number and space symbolically (digits, arrows) rather than physically (e.g. number of dots, screen location). Digits may be more salient to the 5 year-olds than arrows, making them more difficult to ignore and so interfering more with their ability to accurately judge time. In support of this, we found evidence for a developmental dissociation not only in the degree of attentional interference produced by digits and arrows but also in their ability to bias time judgements: the lengthening effect of digits appeared at 8 years old, while the lengthening effect of arrows appeared only from the age of 10 years. This dissociation might reflect the different ages at which children integrate symbolic representations of number (digits) versus spatial position (arrows) or it might suggest the existence of different mechanisms underlying the processing of spatial position versus number. In fact, the dissociation in the lengthening effects of digits versus arrows may provide us with information as to the mechanism underlying these effects. Given that spatial position did not influence time judgements in 8 year olds, but number did, we suggest that the distorting effects of number on time judgements in this age group were not due to a spatial representation of number (mental number line) but, more likely, to general effects of magnitude.

Indeed, prior developmental studies have suggested a spatial representation of time in children as young as 4–5 years old, based on interference effects between temporal and spatial magnitude [37,51]. Very recently, de Hevia et al. [76] have shown that spatial extent and numerical quantity influence duration judgements even in neonates, while Merritt, Casasanto and Brannon [77] have shown that spatial distance influences temporal duration judgements in monkeys. According to these studies, their results suggest the existence of a common underlying representation of magnitude in each of the three dimensions, and could be taken to indicate an innate spatial representation of time, at least in terms of spatial *magnitude*. So why, in our own study, do we fail to see spatial interference of time judgements before 10 years of age? A key difference between these studies and our own is the nature of the spatial representation. While prior studies manipulated the *magnitude* of the spatial representation (i.e. distance), we manipulated its spatial *position* (rightward vs. leftward arrow), either directly using directional arrows, or indirectly using digits. This crucial distinction between spatial representation in terms of magnitude and of position most may explains why prior studies found that spatial information (distance) distorted time judgements in children as young as 5 years old [37], whereas we found no evidence that spatial information (position) influenced time judgements in children of the same age range. In fact, the discrepancy in these results may actually provide useful information as to the developmental trajectory of the spatial representation of time. Although time can be represented independently from space (e.g. in a dedicated internal-clock), there might nevertheless be developmental changes in the way in which time is represented in spatial terms (i.e., development of the spatial representation of time). The spatial representation of time might first be operationalized in terms of general magnitude, such that duration could be represented by distance (or quantity) or, indeed, vice versa [76,77]. However, later in development, the spatial representation of time might then be refined to also include position, such that duration is represented from left to right. Indeed, our own results indicate that the spatial representation of time in terms of position (i.e. the mental time-line) is acquired only around the age of 8–10 years old, at least when position is represented symbolically with arrows.

Another distinction between our own study and those that manipulated space in terms of magnitude (i.e. distance) is that the spatial information derived from arrow cues depends upon an acquired, internalized symbolic representation of space (“endogenous”), whereas the spatial information derived from distance is given automatically and directly by the visuospatial properties of the stimuli themselves (“exogenous”). Numerous studies have demonstrated that judgements of time are acutely sensitive to non-temporal forms of information that are more salient and/or capture more attentional resources than temporal information [78]. Moreover, the interfering effects of non-temporal information on time judgements change as a function of development in attentional capacities [79,80]. Our next step is to directly compare the influence of exogenous and endogenous forms of spatial and number representation on time judgements in children. If results show that non-symbolic representations of spatial position (e.g. lateralized stimulus presentation) or number (e.g. array size) also fail to influence temporal judgements in children younger than 8–10, this would confirm that our current results reflect the development of a mental time-line rather than simply reflecting young children’s inability to interpret symbolic representations of spatial position (i.e. arrows) or number (digits).

For the moment, it is premature to provide a definitive account of the developmental trajectory of spatial and numerical influences on time judgment. There is likely not one but several factors involved in this phenomenon (e.g. magnitude effects; mental number/time lines; endogenous versus exogenous representations; development of attentional resources; brain development), and further experiments are required to better understand the role of each factor [20]. Nevertheless, our results provide convincing evidence that the presence of non-temporal information interfered with time judgments in all participants, even the youngest ones, but that the time *distortions* induced by symbolic representations of space or number did not occur early in development but, instead, emerged with age, between 8 to 10 years old. In conclusion, time distortions produced by a left-right spatial representation of time (i.e. a mental time-line) appear to result from processes acquired throughout childhood. However, the manner by which these spatial representations of time have been learned is still unknown. They could have been learned through education (cultural acquisition of a spatial representation of time) or, alternatively, learned by direct experience of correlations in environment between number, space and time [77]. Several experiments are now required to examine the role of attention and learning in the emergence of spatial and numerical interference of time perception and the complex time-space-number interaction. The key question is, how do children learn to travel mentally in time?

## Supporting Information

S1 File Datasets of the proportion of “long” responses, Bisection Point and Weber Ratio for each participant in each experimental condition in Experiments 1, 2.1 and 2.2. (XLSX)
